# Muscle spindle receptors and their impact on Parkinson´s disease and Cerebral Palsy subjects

**DOI:** 10.1007/s10974-024-09682-8

**Published:** 2024-11-14

**Authors:** Else Marie Bartels, Adrian Harrison

**Affiliations:** 1 Department of Neurology, Copenhagen University Hospital, Bispebjerg and Frederiksberg, Copenhagen, Denmark; 2 PAS, Section for Physiology, Department for Veterinary and Animal Sciences (IVH), Faculty of Health & Medical Sciences, University of Copenhagen, Dyrlaegevej 100, Frederiksberg C, 1870 Denmark

**Keywords:** Muscle spindles, Stretch receptors, muscle, Mechanoreceptors, Muscle tonus, Parkinson´s disease, Cerebral Palsy

## Abstract

In some neurological conditions, like Parkinson’s disease (PD) and Cerebral Palsy (CP), as well as with ageing, muscle spindles have been mentioned as participating in the pathological response of observed muscles. The aim of this review has therefore been to examine what is known about muscle spindle receptors, their function and how they are involved in regulating precise muscle movement in relation to these two conditions. Data from acoustic myography (AMG) studies with healthy controls (HC), CP and PD subjects have been re-examined with a view to identifying possible effects of changes in muscle movement which could be related to muscle spindle receptor function. Studies of muscle spindles have shown that during shortening and lengthening contractions the fusimotor system is activated differently with different discharge frequencies and sensitivities. With increasing age comes a loss of precise proprioception, something that coincides with a change in the AMG E-score towards lower values, indicating a reduced level of coordination and efficiency of muscle use. With PD and CP there is likewise a documented decrease in proprioception, also showing lower E-values than age-matched HC subjects. We conclude that the decrease in proprioception observed in these subjects must be partly due to a change in the muscle spindle / C-centre feedback system.

## Introduction

The aim of this review has been to re-examine the potential role played by muscle spindles in the muscle changes experienced by two neurological conditions, Parkinson´s disease (PD) and Cerebral Palsy (CP). In recent years, a novel approach of acoustic myography has evolved and been tested in clinical settings, where it has been shown to reveal muscle function changes in such conditions as PD and CP (Celicanin et al. 2023; Pingel et al. 2019). The method of acoustic myography does not measure muscle force, but instead records the spatial- and temporal-summation of muscles whilst physically active, as well as the degree of efficiency/coordination (Bartels et al. 2020; Harrison 2017). Two of the parameters, however, have been shown to be very closely correlated with maximal voluntary force (Claudel et al. 2019).

Recently it was shown that for the biceps and extensor carpi radialis longus muscles of PD patients active movement resulted in a lower spatial to temporal ratio signal (S: T ratio) relative to healthy controls, yet no significant difference between the patient and control groups was noted for the triceps muscle (Celicanin et al. 2023). These measurements were carried out with acoustic myography (AMG). In the afore mentioned study, Celicanin and colleagues reported that the main finding was that the S: T ratio changed from being principally temporal summation (higher motor unit firing rate) in the healthy control group (HC), to being primarily spatial summation (more motor units active) in the PD group, in *m. biceps brachii* and *m. extensor carpi radialis longus* – both of which are flexor muscles (Celicanin et al. 2023). Flexors are muscles that primarily decrease the angle of a joint, whilst extensors do the opposite and increase the angle of a joint. Celicanin and colleagues likewise noted that *m. triceps brachii* did not show any differences in AMG scores between the two subject groups during elbow movements (Celicanin et al. 2023).

This interesting difference between m. biceps brachii and *m. triceps brachii* in PD patients may be indicative of an underlying neural innervation difference between flexors and extensors. This thought is supported by the finding of an improvement in resting elbow joint angle following medication (Marusiak et al. 2018), and favouring the idea that rigidity is present to a higher degree in flexors than in extensor muscles in PD subjects, possibly as a result of a higher resting tone of the flexor muscles cf. extensors (Andrews et al. 1972).

Another neurological condition that affects muscle function is CP. AMG recordings have shown that CP subjects have a significantly lower initial S-score (spatial summation) than that of healthy matched controls. Neither the T-score (temporal summation) or the E-score (efficiency) were found to be different from the healthy control group. The results point to a change in the recruitment of skeletal muscle fibres with CP, such that affected subjects use a higher degree of spatial summation (more fibres recruited) to maintain the same treadmill performance as that of the controls (Pingel et al. 2019).

## Muscle spindle function

Muscle spindles are specialized modified muscle fibres that act as sensory receptors, sending information about muscle length, speed of stretch as well as perceived effort to the central nervous system. The combined information provided by muscle spindle receptors is then used to determine the position and movement of our joints (proprioception). As muscle spindles react to changes in muscle length, they are also involved in the regulation of muscle contraction (Santuz and Akay 2023).

Of considerable interest are the older findings of Burke and colleagues, who looked into muscle spindle function. They noted that during shortening contractions the fusimotor system is activated together with the skeleto-motor system (Burke et al. 1978). However, the fusimotor drive is generally insufficient to maintain a significant spindle discharge unless movement is slow, or the muscle is shortening against an external load (isotonic contraction) (Burke et al. 1978). These authors also noted that during lengthening contractions (eccentric) the spindle responses were greater than observed during passive stretch of a similar amplitude and velocity, suggesting heightened fusimotor outflow. Indeed, they were able to measure the mean discharge from a dynamic spindle ending in the tibialis anterior muscle during voluntary movement to be in the order of 7.5 per second during stretch, but only 1.0 per second during shortening, a difference that disappeared with loading (Burke et al. 1978).

These findings raise the question as to whether the motor activity of flexion or extension results in different firing rates for the muscle spindles embedded within these muscles. One potentially important consequence of such a difference would be to have a protective effect, sending frequent signals back to the central nervous system (CNS) every time a muscle is extended whilst contracting, presenting a risk of damage, yet sending relatively few signals when a muscle is shortened under contraction, simply because the risk of injury is so much less with such motor activity.

**Fig. 1:**
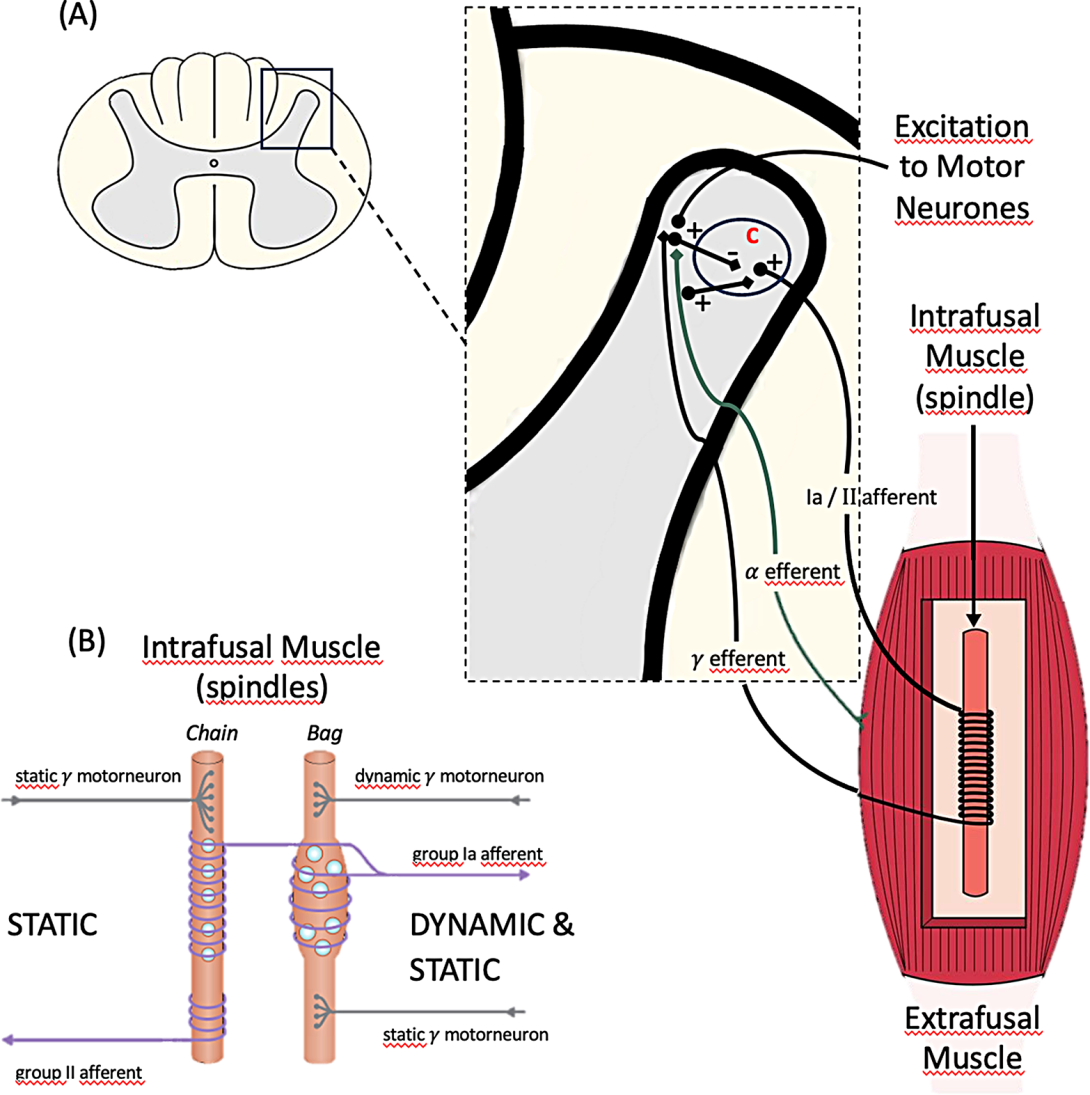
An overview of intrafusal muscle spindles. (**A**) illustrates the link between extrafusal and intrafusal innervation, including 𝛼 efferent motor neurone innervation to extrafusal fibres and 𝛾 efferent innervation to the muscle spindles. Note also the Ia / II afferent innervation from the muscle spindles back to the CNS and more specifically to a region we have referred to as C (Control Centre; see (McClusky et al. 1983) where there is both a negative stimulus from the 𝛾 efferent neurone and positive stimulus from the Ia / II afferent neurone. (**B**) provides an overview of the types of static and dynamic muscle spindles found associated with extrafusal fibres and their efferent and afferent innervation. This figure does not illustrate the $\:\beta\:\:\text{m}\text{o}\text{t}\text{o}\text{r}\text{n}\text{e}\text{u}\text{o}\text{n}\text{e}\:\text{e}\text{f}\text{f}\text{e}\text{r}\text{e}\text{n}\text{t}\:\text{f}\text{i}\text{b}\text{r}\text{e}\:$innervation. This innervation comprises combined static/dynamic fibres showing both extrafusal and intrafusal fibres. γ motorneurones have the sole function of addressing fusi-motion. Spindle α motorneurones are solely extrafusal fibre related and are skeleto-motor in function. $\:\beta\:\:$motorneurones can be skeleto-motor and fusi-motor, but never fusi-motor alone. (Source & Copyright EMB & AH)

An overview of a typical muscle spindle is given in Fig. 1.

The C marked on panel (A) of Fig. 1 represents a Control Centre in the region of the spinal cord (location yet to be determined by histology) that senses Ia / II afferent signals and integrates them with signals from the fusimotor system (𝛾 efferent and probably also $\:\beta\:$ efferent), such that any signal in excess of the fusimotor signal constitutes a kinesthetic signal (McClusky et al. 1983). Various models have been proposed for the function of the region we refer to as C, such as a Signal Processing Area facilitating sensory motor performance in a task specific way and not only a measurement of posture and movement (Dimitriou and Edin 2010). Another model presents Forward Mediated Processing, created by interaction changes in length of muscle spindles and their adaptive response, used for perception of effort (Monjo and Allen 2023). Others have even suggested that there is evidence for the abundance of muscle spindles in regulating muscle contraction (Kissane et al. 2022). Kinesthetic signals are more commonly known today as proprioception, a system whereby feedback sensations relating to body position, movement and the position of body parts relative to each other are combined with muscle contractions. One could even speculate that stiffness or contracture in a muscle may to some extent be due to a mismatch between spindle signals arriving at the C-cell. Furthermore, the motor excitation signal as well as any other regulatory signal arriving at the C-cell, may override part of, or all of the spindle signal, and together these signals determine the degree of overall muscle contraction.

Intrafusal muscle spindles are stretch receptors whose function is to correct for changes in muscle length when extrafusal muscle fibres are either shortened (under contraction) or lengthened (under stretch). Such muscle spindles and their reflexes operate to return extrafusal muscle to its resting length after it has been shortened or lengthened, as well as to maintain length-tension at its optimal setting (Winters et al. 2011). In terms of sensory innervation, each intrafusal muscle spindle consists of either a Ia or II afferent nerve, which innervates the central region of the nuclear bag fibres (dynamic/static: Ia) or the nuclear chain fibres (static: II) (see Fig. 1B). It is important to note that the Ia fibres are among the largest nerves in the body with some of the fastest conduction velocities (80–120 m/s), whilst II fibres have intermediate diameters and intermediate conduction velocities (33–75 m/s). Motor innervation of the intrafusal muscle spindles consists of two types of γ motor neurones (both dynamic and static), where dynamic γ motor neurones synapse on the nuclear bag fibres, and static γ motor neurones synapse on nuclear chain fibres which spread over longer distances. It is worth noting that γ motor neurones are smaller and slower than the α motor neurones that innervate the extrafusal fibres, and they serve to regulate the sensitivity of the intrafusal muscle fibres they innervate (Prochazka and Hulliger 1983).

In the feline it has been shown that $\:\beta\:\:$motor neurone fibre stimulation is widespread and functionally significant. Moreover, spindle morphology in man points towards great similarity between spindle function in the cat and rat (Prochazka and Hulliger 1983) suggesting that $\:\beta\:\:$motor neurone fibre stimulation may also be present in man, and could perhaps be a common mammalian feature?

The afferent signal from muscle spindles is generally classified as being both by a primary (Ia) and a secondary (II) motorneurone based on their differences in their dynamic sensitivities to stretch (Hagbarth 1993). At resting length discharge rates for muscle spindles are often measured to zero, and during stretch at a constant level of force the discharge rate has been shown to be typically less than 25 per second (25 Hz). Under conditions where subjects voluntarily applied the fastest stretch possible, the peak rate of discharge was less than 100 Hz and more often less than 50 Hz (Prochazka and Hulliger 1983).

Position sensitivity for muscle spindles and the afferent signal from relaxed muscles is very low (0.18–0.28 Hz) per degree of joint movement (Prochazka and Hulliger 1983). Some loss of sensitivity may occur as a result of the known decrease in the number of intrafusal fibres as well as the increase in capsular thickness, and signs of denervation in some spindles, all of which occur with increasing age (Swash and Fox 1972; Liu et al. 2002). Findings that lend support to a loss of sensitivity in muscle spindles with increasing age causing the known decrease in accurate proprioception (Lord et al. 2019).

With PD, Camptocormia, which is an axial thoracolumbar deformity, may result in a bent back angle (Schulz-Schaeffer 2016; Bloch et al. 2006). For this to happen, the sensory input from muscle spindles and joint receptors which gives a sense of joint position, of movement and the sense of muscle strength, is most likely compromised. In PD an impaired proprioception has been demonstrated and could be part of the underlying cause for occurrence of camptocormia (Bloch et al. 2006). Keeping in mind that the muscle spindle system is mature already at a young age (Österlund et al. 2011), and that PD patients typically demonstrate perfect healthy motoric skills during a substantial part of their life, this raises the question as to whether and indeed when changes in spindle activity and possibly structure take place with PD. This could, moreover, provide an early diagnosis for this disease. Accepting the hypothesis that muscle spindles participate in regulating muscle contraction, then AMG measurements of suspected CP or PD individuals where the clinical studies to date show a change in the S: T to values less than 1.0 (Celicanin et al. 2023; Pingel et al. 2019), might prove a useful technique for measuring this (see below, Table 2).

Furthermore, it would be of particular value if it was possible to slow down the development of such proposed changes in the PD spindle system. In healthy subjects exposed to eccentric exercise to the point of fatigue, muscle spindles still give the correct information concerning limb position, although some dynamic position error occurs (Celicanin et al. 2023). This would mean that whilst the angle of contraction of a joint is retained, an element of inaccuracy is introduced whilst the limb is in movement.

With advanced PD the responses to mechanical vibrations of the Ia afferents become compromised, and this is not improved by medication such as L-3,4-dihydroxyphenyalanine known medically as levodopa DOPA (Valkovic et al. 2006). This change may further be linked to the growing dependency on visual inputs to carry out certain types of movement as the disease progresses (Lee 1989), and the precision with which movement and location of the limb relative to the joint are compromised. The precise signalling behind this change is still not clear, but they might be a further development of the phenomenon associated with changes in Ia signalling (dynamic: bag fibres). In support of the findings with PD patients, it has been found in the cat that DOPA administration resulted in an enhanced muscle spindle activity in the static state for both flexor and extensor muscles, yet in the dynamic state the activity of flexor muscles was depressed (Prochazka 1981; Johansson et al. 1995). Furthermore, it has been shown that autogenetic reflex effects from large muscle afferents are relatively weak in the spinal cat treated with DOPA (Prochazka 1981). This is in accordance with the difference in diameter of the afferent nerves (Ia vs. II).

### Acoustic myography (AMG) data and possible connection to PD spindle behaviour

Focusing on possible changes in the function of muscle spindles in PD, the AMG data from Celicanin et al. (2023) tell a story on their own. The AMG data in Table 1 are directly taken from the Celicanin et al. (2023) study. The AMG parameters are efficiency (E-score), spatial- (S-score) and or temporal-summation (T-score) given by the E-, S-, and T-scores. As an overall measure of muscle function, the combined mean EST score (Mean EST = E-score + S-score + T-score / 3) is shown. It is interesting to note that L-DOPA administration to PD subjects improves their overall muscle function (see Table 1; Mean EST). However, the PD patients do not reach the same level of mean EST as the healthy controls, whether this be for passive or active movements. Indeed, the mean EST values typically reach a level of 66–75% of the healthy controls. Clearly then the discrepancy must be with an altered efficiency, spatial- and or temporal-summation (E-, S-, T-score). For the m.Triceps, both for passive and active activity, the discrepancy is found to be a combination of all three parameters, which remain lower than comparable values for healthy matched controls.

**Table 1:** Additional calculations, using the data in (Celicanin et al. 2023). The AMG parameters E- S- and T- plus the mean EST, as well as S: T and the relation between Passive: active (P: A) for S, T, and E, are given for healthy age matched control subjects (HC) and Parkinson´s disease subjects (PD) for muscles triceps and biceps for both passive and active movements. Mean age of the healthy controls was 68.5 ± 7.3 years and of the PD subjects was 68.5 ± 7.3 years. PD0 are data from PD who have not received medication, PD3 are data from PD who received L-DOPA three hours before measurement.Values are means ± standard deviation

Muscle	Movement	E-score	S-score	T-score	S: T	Mean EST	Explanation PD vs. C
*m.Triceps*	Passive	HC 5.0±2.5 PD0 2.3±3.1 PD3 2.1±2.4	HC 9.2±0.4 PD0 5.4±3.6 PD3 7.0±2.6	HC 6.5±2.2 PD0 4.5±1.7 PD3 4.7±2.2	1.4 1.2 1.4	6.9 4.0 4.6	Increased spatial and temporal summation, lower efficiency with PD0
	Active	HC 4.4±2.4 PD0 1.6±3.0 PD3 1.9±2.6	HC 8.8±0.5 PD0 4.7±3.4 PD3 4.9±3.4	HC 4.8±2.3 PD0 4.6±1.8 PD3 5.2±1.9	1.8 1.0 0.9	6.0 3.6 4.0	Increased spatial summation and lower efficiency with PD0
	P: A	HC 1.1 PD0 1.4 PD3 1.1	HC 1.0 PD0 1.1 PD3 1.4	HC 1.3 PD0 0.9 PD3 0.9	-	-	Mostly similar Passive to Active ratio for C and PD0 for S-score
*m.Biceps*	Passive	HC 2.6±1.9 PD0 0.9±1.2 PD3 2.0±2.2	HC 8.6±1.2 PD0 3.7±3.3 PD3 5.4±3.4	HC 5.2±1.8 PD0 4.5±1.7 PD3 4.6±1.4	1.6 0.8 1.1	5.4 3.0 4.0	Increased spatial and temporal summation, lower efficiency with PD0
	Active	HC 2.4±1.1 PD0 0.4±0.4 PD3 1.3±2.1	HC 7.3±2.2 PD0 3.4±3.3 PD3 4.0±3.6	HC 3.8±1.4 PD0 4.9±2.4 PD3 5.1±1.9	1.9 0.7 0.7	4.5 2.9 3.4	Increased spatial summation and lower efficiency with PD0
	P: A	HC 1.1 PD0 2.2 PD3 1.5	HC 1.2 PD0 1.1 PD1 1.3	HC 1.3 PD0 0.9 PD1 0.9	-	-	Mostly similar Passive to Active ratio for HC and PD0 for S-score

It is also interesting to note that for m.Triceps the passive to active ratio (P: A) returns to healthy control values for the E-score, whilst the S-score ratio is higher and the T-score ratio lower than that of healthy matched controls, indicating a persistent altered function, but one that most likely does not affect muscle spindles.

Despite the fact that PD patients seem to have a normal muscle function following medication, the EST mean reveals that they are over-working their muscles compared to age-matched controls. This is the case for both the extensor and the flexor muscles. Medication clearly improves the PD condition, but the treatment does not restore muscle function to the same level as that found in HC. The E-score following medication is the same for PD as for HC, whilst the S-score is higher and the T-score lower than that of HC, indicating a persistent altered function.

For the *m.Biceps* and passive activity the discrepancy is likewise to be found as a combination of all three AMG parameters, which remain lower than comparable values for the HC group, yet under active movements this muscle shows a lower E- and S-score, but a higher T-score. It was noted that for *m.Biceps* the passive to active ratio (P: A) returns to HC values for the S-score following medication, whilst the E-score is higher and the T-score lower than that of healthy matched controls, also indicating a persistent altered function. In consideration of these findings, one could suggest that for *Biceps* the change in P: A for the E-score most likely indicates a spindle effect, which could represent the chain fibres working normally but the bag fibres (dynamic) being partly disrupted. Interestingly, such changes are also observed with fatigue, as mentioned earlier (Grose et al. 2022).

## Acoustic myography (AMG) data and possible connection to CP spindle behaviour

When comparing the PD data with Cerebral Palsy (CP) data from Pingel et al. (2019), it was found that the E-score in CP was the same as that in the healthy controls (HC) group. The S-score revealed a significant difference between the CP subjects, who show a lower initial score, compared with the HC group, who had a higher initial score (*P* < 0.01 to *P* < 0.05). This finding indicates that the CP subjects have a higher degree of spatial summation to maintain the same speed of treadmill activity than the control subjects, i.e. they were recruiting significantly more fibres than the controls. The T-score was found to be the same for the CP and the HC group. Initially, the T-score of 8.0, which equates to a firing frequency of 51 Hz, was found after 30 min of treadmill exercise to change to a T-score of 7.1, which equates to a firing frequency of 73 Hz. The actual S-score values for the CP subjects were; 6.25 ± 0.74, 6.03 ± 0.84 and 5.94 ± 0.96 at 1, 5 and 9 min of exercise, respectively, compared with values of 7.42 ± 0.80, 7.34 ± 0.81 and 7.29 ± 0.84 for the healthy controls at the same times.

**Table 2:** Additional calculations, using the data in (Pingel et al. 2019). The AMG parameters E- S- and T- plus the mean EST, as well as S: T for *m. gastrocnemius* are given for CP subjects. The subjects were required to participate in a single treadmill session where they were asked to walk or run as fast as they could for a period of 30 min. For further details see Pingel et al. (2019). Values are Mean ± SD (*n* = 10)

Exercise (mins)	1	3	5	7	9	11	13	15	17	19	21	23	25	27	29
E-score	4.99 ±1.45	4.64 ±1.50	4.38 ±1.64	4.28 ±1.58	4.19 ±1.32	4.16 ±1.54	3.73 ±1.48	3.63 ±1.37	3.68 ±1.10	3.26 ±1.10	3.23 ±1.62	3.25 ±1.81	3.15 ±1.55	3.19 ±1.46	3.13 ±1.74
S-score	6.25 ±0.74	6.05 ±0.78	6.03 ±0.84	5.93 ±0.97	5.94 ±0.96	5.84 ±0.93	5.82 ±0.97	5.78 ±1.13	5.76 ±0.98	5.61 ±1.20	5.54 ±1.24	5.43 ±1.38	5.43 ±1.33	5.29 ±1.41	5.33 ±1.60
T-score	7.84 ±0.64	7.54 ±0.94	7.53 ±0.89	7.01 ±1.96	7.30 ±1.47	7.27 ±1.10	7.18 ±1.27	7.28 ±0.98	7.11 ±0.88	7.14 ±0.91	7.06 ±1.30	7.06 ±0.63	7.00 ±0.80	6.98 ±0.78	7.15 ±0.55
Mean EST	6.36	6.08	5.98	5.74	5.81	5.76	5.58	5.56	5.52	5.33	5.28	5.24	5.19	5.16	5.20
S: T ratio	0.80	0.80	0.80	0.85	0.81	0.80	0.81	0.79	0.81	0.79	0.79	0.77	0.78	0.76	0.75

The results for both PD and CP subjects suggest that the S: T ratio in both groups is affected such that values fall below 1.0. This makes them different from healthy controls where the S: T value is always greater than 1.0 (typically 1.4 or higher, except during exercise to exhaustion). This change in the S: T arises because the T-score is higher than the S-score. In CP the T-score does not differ significantly from that of the healthy controls throughout the period of exercise. The significant difference between the S-score of the CP subjects and that of the controls was only seen for the first 10 min of exercise. Following this, the healthy controls showed exhaustion and an S: T below 1.0 (Pingel et al. 2019).

## Discussion

Whilst a lot remains unknown about muscle spindle receptors, their regulation and control, there is now evidence from clinical and animal studies that implicates them in normal muscle function, as well as the diseased state.

Regulation of muscle contraction with PD, involves fine motor control, rigidity, tremor and a loss of proprioception. AMG recordings for PD detect a lower E-score, which means that muscles are active more of the time for any given function compared with a HC group of the same age, most likely inducing early fatigue as a consequence. This is in accordance with our earlier study (Bartels et al. 2020) which found the following E-score values for young *versus* older healthy subjects: *Biceps* flexion: E-score for HC aged 20–29 = 5.9±2.4 vs. 60–69 = 2.4±1.1; *Triceps* flexion: E-score for HC aged 20–29 = 6.9±2.1 vs. 60–69 = 4.4±2.4; *Gastrocnemius* cycling minimal load: E-score for HC aged 20–29 = 6.6±1.9 vs. 60–69 = 1.4±1.3. The finding for this change in E-score for both PD as well as HC with aging indicates that the regulation of muscle contraction is not optimal, pointing towards impaired coordination/efficiency of muscle use, which may involve the spindle system. One could speculate that such a compromise to the muscle occurs at the C-cells and the interpretation of incoming Ia / II afferent signals in relation to incoming motor neurone signals, resulting in a loss of smooth and efficient muscle contraction.

It has been found that vibration applied to the body has a beneficial effect on muscle balance (Cochrane 2011; Volpe et al. 2014). Cochrane used vibration platforms or small devices intended to vibrate tendons or other body parts, for example handheld vibrating dumbbells (0–30 Hz), or vertical sinusoidal vibration (SV) devices used during dynamic movement (joint movement 5-35^o^; SV forward 5–65 Hz). In PD muscle, tremor (range 5–10 Hz) is a typical symptom and it is relatively resistant to attempts to reset it using mechanical perturbation (Lee and Stein 1981). Perhaps this may be due to altered feedback from muscle spindles, for example Ia (bag fibres) and/or C cell inhibition or damping? The AMG results for PD and older healthy subjects points towards a less efficient muscle use (low E-score) which very likely could be an effect of altered function of the chain fibres. Such a change could be expected to give a subject an impaired perception of the true starting position of any given muscle, as well as an incorrect assessment of effort. A knock-on effect would be that the bag fibres incorrectly perceive the effort needed and compensate by recruiting more fibres than necessary (seen as a low AMG S-score). This would also contribute to earlier signs of fatigue.

In support of the AMG findings, altered settings of muscle spindles in CP subjects were suggested previously (Mockford and Caulton 2010). Spindles most likely also play a role in spasticity, since they record changes in muscle length and velocity in CP subjects during passive stretches and during normal gait (Falisse et al. 2018). Peripheral sensory neuropathy in CP may therefore be due to chronic overload of afferent impulses from muscle spindles in these spastic muscles (Fukuhara et al. 2010) once again implicating the C-cell region. With CP the AMG signal has an E-score and T-score that are identical with that of the HC group, whilst the S-score is significantly lower, indicating that they recruit more muscle fibres (spatial summation) for the same task (Pingel et al. 2019). It may be suggested that the spasticity in these muscles is in some way related to the spindle´s static sensing (chain fibres) and perhaps also their dynamic sensing (bag fibres). The need to recruit more fibres for any given task (AMG S-score), indicates a need to overcome some initial inertia or resistance, which might be due to a changed function of the chain fibres, but the fact that they remain different with a lower S-score (more active fibres) also implicates involvement by the bag fibres. Although the CP and PD subjects represent very different conditions, they may be linked through an altered muscle spindle function. One could speculate that muscle spindle receptors become altered in their function as a result of an increased perception of effort, that is disproportionate to the actual requirements for any given physical task, as seen with PD and CP.

## Conclusion

There is still a great need for more information concerning the regulatory roles of muscle spindle receptors, as well as the proposed role of a Control-centre for spindle regulation in the CNS, which remains unidentified. Recent acoustic myography data combined with the available data from existing muscle spindle studies indicate that with CP or PD, muscles not only fatigue more quickly (lower AMG ST value) than those of healthy subjects under identical conditions, they also indicate signs of decreased proprioception (lower AMG E-score). This can only be due to a change in the spindle signal / Control-centre feedback system, perhaps especially involving the chain fibres, having a knock-on effect on the bag fibres. Overall, the consequence of these changes is an altered perception of the amount of physical effort needed to successfully complete any given muscle task, with a disproportionate recruitment of muscle fibres (low AMG S-score) and a tendency to activate muscle fibres for a longer period of time than actually required (low AMG E-score) giving rise to premature muscle fatigue.

## Data Availability

No datasets were generated or analysed during the current study.
